# Risk factors for adverse outcomes in vaginal preterm breech labor

**DOI:** 10.1007/s00404-020-05731-y

**Published:** 2020-08-07

**Authors:** Anna Toijonen, Seppo Heinonen, Mika Gissler, Georg Macharey

**Affiliations:** 1 Department of Obstetrics and Gynecology, University Hospital (HUS), University of Helsinki, Haartmaninkatu 2, 00290 Helsinki, Finland; 2 National Institute for Health and Welfare (THL), Helsinki, Finland

**Keywords:** Preterm delivery, Preterm labor, Breech presentation, Vaginal labor, Risk factor, Adverse outcome

## Abstract

**Purpose:**

To assess the risk factors for adverse outcomes in attempted vaginal preterm breech deliveries.

**Methods:**

A retrospective case–control study, including 2312 preterm breech deliveries (24 + 0 to 36 + 6 gestational weeks) from 2004 to 2018 in Finland. The preterm breech fetuses with adverse outcomes born vaginally or by emergency cesarean section were compared with the fetuses without adverse outcomes with the same gestational age. A multivariable logistic regression analysis was used to calculate the risk factors for adverse outcomes (umbilical arterial pH below 7, 5-min Apgar score below 4, intrapartum stillbirth and neonatal death < 28 days of age).

**Results:**

Adverse outcome in vaginal preterm breech delivery was associated with maternal obesity (aOR 32.19, CI 2.97–348.65), smoking (aOR 2.29, CI 1.12–4.72), congenital anomalies (aOR 4.50, 1.56–12.96), preterm premature rupture of membranes (aOR 1.87, CI 1.00–3.49), oligohydramnios (28–32 weeks of gestation: aOR 6.50, CI 2.00–21.11, 33–36 weeks of gestation: aOR 19.06, CI 7.15–50.85), epidural anesthesia in vaginal birth (aOR 2.44, CI 1.19–5.01), and fetal growth below the second standard deviation (28–32 weeks of gestation: aOR 5.89, CI 1.00–34.74, 33–36 weeks of gestation: aOR 12.27, CI 2.81–53.66).

**Conclusion:**

The study shows that for each subcategory of preterm birth, there are different risk factors for adverse neonatal outcomes in planned vaginal breech delivery. Due to the extraordinary increased risk of adverse outcomes, we would recommend a planned cesarean section in very preterm breech presentation (28 + 0 to 32 + 6 weeks) with severe maternal obesity, oligohydramnios, or fetal growth restriction and in moderate to late preterm breech presentation (33 + 0 to 36 + 6 weeks) with oligohydramnios or fetal growth restriction.

## Introduction

Around 4% of all fetuses are in breech presentation at birth [1, 2]. In preterm labor breech presentation is more common than in term and every fourth of all fetuses born extremely preterm are in breech presentation at birth [3–6]. Breech presentation in preterm and term pregnancies is associated with obstetric risk factors for adverse neonatal outcomes, such as oligohydramnios, fetal growth restriction, and congenital anomalies [7–9]. Vaginal breech delivery at term is a risk factor for short-term neonatal morbidity [10], and therefore, vaginal breech delivery is feasible only for well-selected patients [11–15]. In vaginal preterm breech delivery, the situation is still unclear in which cases a vaginal delivery is associated with an increased adverse neonatal outcome. The royal college of obstetricians and gynaecologists stated in their breech delivery guidelines that a spontaneous vaginal breech labor in preterm pregnancies is not contraindicated if an immediate cesarean delivery is not needed for maternal or fetal reasons [16]. In many countries, cesarean section is the most common way of delivery for preterm breech fetuses as several studies have suggested that preterm breech fetuses delivered by a primary cesarean section have a significantly lower risk of neonatal mortality compared with those delivered vaginally [17–19]. Cochrane review 2013 could not recommend the mode of birth instead of another in preterm deliveries irrespectively of fetal presentation [20].

Earlier studies were able to identify risk factors for adverse neonatal outcome in vaginal term breech deliveries [8, 11, 21], but there is no research available regarding risk factors for adverse neonatal outcome in vaginal preterm breech delivery. Our study aims to identify risk factors for adverse neonatal outcomes in vaginal preterm delivery. This information is needed, since every tenth baby is born preterm [20] and many of them are in a breech position. Obstetricians need adequate information to identify those women who should give birth by cesarean section in any case.

## Materials and methods

The study is a population-based case–control study, including all singleton breech deliveries from 24 to 36 completed weeks of gestation that were delivered vaginally or by emergency cesarean section in Finland. The study period ranged from January 1st, 2004 to December 31st, 2018. The population included altogether 2312 preterm breech deliveries.

We utilized the data of the national medical birth register and the hospital discharge register maintained by the Finnish Institute for Health and Welfare. The authorization to use the data was obtained from the Finnish Institute for Health and Welfare as required by the national data protection law in Finland (reference number THL/652/5.05.00/2017). All maternity hospitals are obligated to report clinical data on national registers. The national medical birth register includes all live births and stillbirths from 22 weeks or from 500 grams. The hospital discharge register contains information on all inpatient and outpatient care in public hospitals including data from maternal, obstetric, and neonatal care. International Statistical Classification of Diseases and Related Health Problems 10th Revision, ICD-10, is used to code the information.

We limited the study population to the fetuses born vaginally or by emergency cesarean section, as we wanted to research intrapartum risk factors of preterm breech deliveries. We excluded multiple gestations, antepartum stillbirths, deliveries before 24 weeks of gestation, and term pregnancies from our study. The deliveries complicated with uterus rupture (ICD-10 O71.0, O71.1), placental abruption, and severe congenital anomalies such as chromosomal and heart defects diagnosed at birth hospital (ICD-10 Q90–Q99, Q20, Q22, Q28) that might have affected on the newborns surviving, were excluded from the study.

The primary outcome of the study was an adverse outcome defined as the following: umbilical arterial pH below 7, a 5-min Apgar score below 4, intrapartum stillbirth or neonatal death between 0 and 27 days of age. The following maternal variables were included in the analysis: age, parity, pre-pregnancy body mass index (BMI), smoking, in vitro fertilization, history of cesarean section, and maternal hypo- or hyperthyroidism (ICD-10 E03, E05). The obstetric risk factors assessed in the analysis were: maternal gestational diabetes (ICD-10 O24.4) and other diabetes treated with insulin (ICD-10 O24.0), arterial high blood pressure or preeclampsia (ICD-10 O13, O14), oligohydramnios (ICD-10 O41.0), and preterm premature rupture of membranes (PPROM) (ICD-10 O42). Induction of labor and the use of epidural anesthesia during labor were also included in the variables. The fetal factors such as sex, fetal birthweight below the second standard deviation (SD), and congenital fetal anomalies, as defined in the register of congenital malformations, were included in the analysis.

We divided the study population into three groups according to the World Health Organization (WHO) definitions of preterm deliveries. A fetus born alive before 37 completed weeks of pregnancy is defined as preterm birth, according to WHO. WHOs recommended subcategories based on gestational age were used in the division of the groups: extremely preterm (less than 28 pregnancy weeks), very preterm (28 to 32 pregnancy weeks), and moderate to late preterm (32 to 37 pregnancy weeks).

The preterm breech fetuses with adverse outcomes were compared with the fetuses without adverse outcomes with the same gestational age. Each study group, divided according to WHO classification, was separately adjusted.

We used SPSS for 19 to perform the statistical analyses. The adjustments with a binary logistic regression model were calculated for the study population. A Chi squared test or Fisher’s exact test was used when appropriate. Odds ratio (ORs) and corresponding 95% confidence interval (CIs) for each risk factor for adverse outcomes were calculated, and *p* values below ≤ 0.05 were considered statistically significant.

## Results

**Fig. 1:**
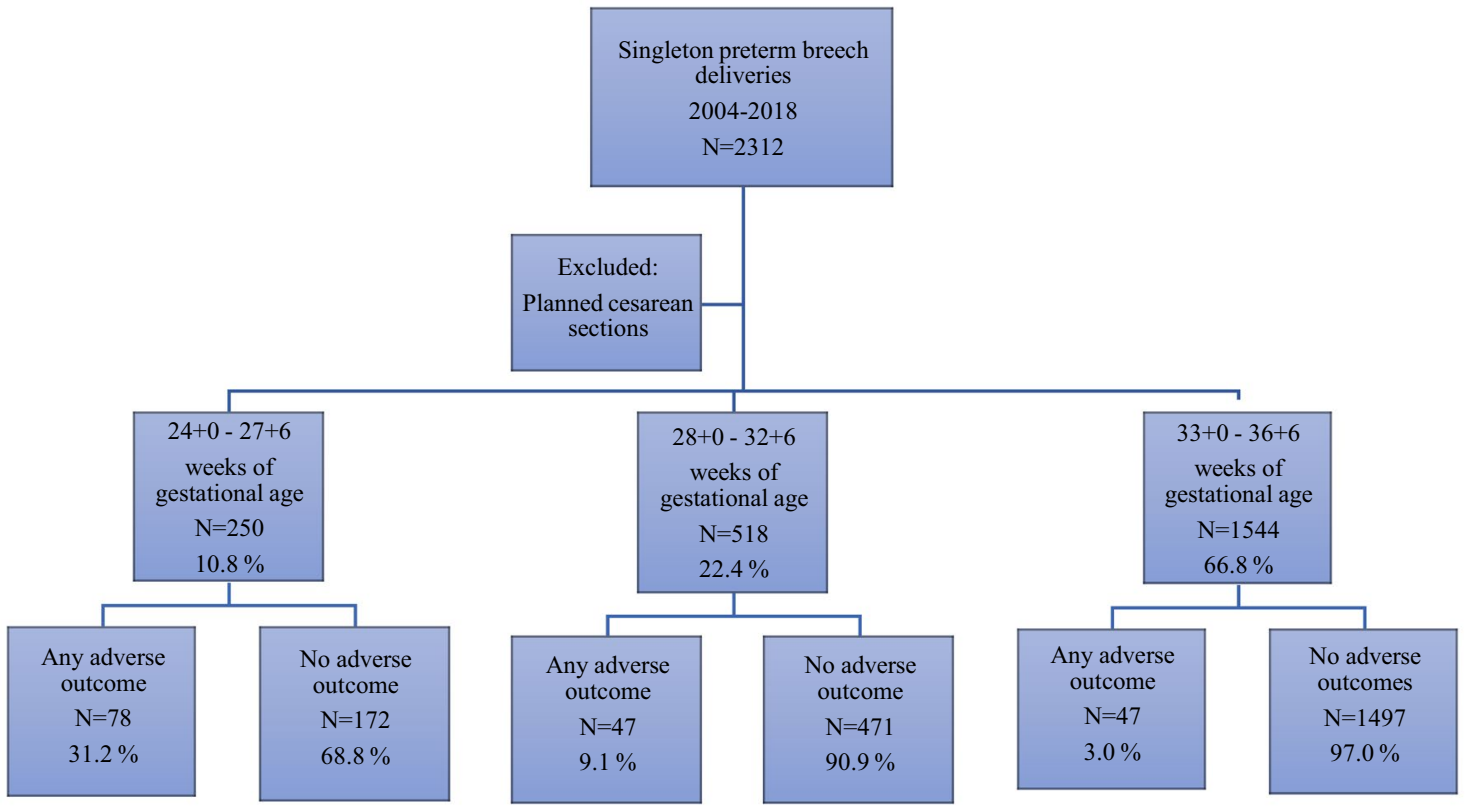
Breech presentation and adverse outcomes during the period of 2004–2018 in Finland

Our study included 2312 singleton preterm breech deliveries born between 24 + 0 and 36 + 6 gestational weeks in 2004–2018 in Finland. Out of these deliveries, 7.4% (172 fetuses) had adverse outcomes. The risk of having adverse outcomes was over tenfold in the fetuses born in 24 + 0 to 27 + 6 weeks of gestation and threefold in 28 + 0 to 32 + 6 weeks of gestation, compared to the late preterm breech deliveries (Fig. 1).

In 24 to 28 weeks of gestational age, 78 out of 250 breech deliveries (31.2%) had an adverse outcome. In these gestational weeks, the only significant risk factor in a trial of vaginal breech labor emerging from our study was PPROM (aOR 1.87, CI 1.00–3.49) (Table 1).

**Table 1:** Unadjusted and adjusted odds ratios for risk factors for adverse outcomes in 24 + 0 to 27 + 6 weeks of gestational age fetuses in breech presentations 2004–2018 in Finland

24–27 weeks of gestation	No adverse outcomes (*N*/%)	Any adverse outcome (*N*/%)	Adjusted OR	95% confidence limits
	*N *= 172	*N *= 78		
Maternal age < 25	5/2.9%	4/5.1%	2.04	0.48–8.66
Maternal age ≥ 35	48/27.9%	23/29.5%	1.31	0.65–2.58
Maternal smoking	30/17.4%	13/16.7	0.91	0.41–2.04
Nulliparous	76/44.2%	42/53.8%	1.82	0.93–3.54
Multipara ≥ 3 deliveries	25/14.5%	12/15.4%	1.37	0.45–4.23
BMI ≥ 30	25/14.5%	11/14.1%	1.20	0.41–3.50
BMI ≥ 35	12/7.0%	4/5.1%	0.65	0.13–3.16
History of induced abortion	36/20.9%	41/17.9%	0.85	0.39–1.83
History of miscarriage	48/27.9%	29/37.2%	1.64	0.89–3.03
History of cesarean section	18/10.5%	9/11.5%	1.13	0.41–2.56
Assisted reproduction technology	7/4.1%	0/0.0%		
Diabetes mellitus type I	2/1.2%	2/2.6%	0.34	0.04–2.63
Diabetes mellitus type II	0/0.0%	1/1.3%		
Gestational diabetes	10/5.8%	4/5.1%	0.99	0.24–4.12
Pre-eclampsia/high blood pressure	20/11.6%	4/5.1%	0.49	0.15–1.58
Oligohydramnios	4/2.3%	2/2.6%	0.88	0.15–5.34
Placenta previa	3/1.7%	1/1.3%	0.54	0.05–5.99
PPROM	0/0.0%	31/39.7%	1.87	1.00–3.49
Any congenital anomaly	0/0.0%	2/2.6%	1.98	0.29–13.59
Induced delivery	47/27.3%	1/1.3%	0.50	0.04–5.97
Epidural anesthesia in vaginal birth	3/1.7%	3/9.4%	1.18	0.39–3.59
Gestational age at delivery mean in days	184/8	179/8		
Fetal growth restriction (birth weight < 2SD)	0/0.0%	1/1.3%		
Neonatal sex (female)	87/50.6%	34/43.6%	0.75	0.42–1.34

*BMI* body mass index, *PPROM* preterm premature rupture of membranes

Among very preterm breech deliveries (28 + 0 to 32 + 6 weeks of gestation), nearly one out of ten fetuses (47/518, 9.1%) had adverse outcomes. Severe maternal obesity (aOR 32.19, CI 2.97–348.65), oligohydramnios (aOR 6.50, CI 2.00–21.11), congenital anomalies (aOR 4.50, 1.56–12.96), and fetal growth restriction (aOR 5.89, CI 1.00–34.74) increased the risks for adverse outcomes in these gestational weeks. Nulliparity (aOR 0.43, CI 0.18–0.99) and maternal preeclampsia or high blood pressure (aOR 0.21, CI 0.05–0.96) were associated with a decreased risk of adverse outcome (Table 2).

**Table 2:** Unadjusted and adjusted odds ratios for risk factors for adverse outcomes in 28 + 0 to 32 + 6 weeks of gestational age fetuses in breech presentations 2004–2018 in Finland

28–32 weeks of gestation	No adverse outcomes (*N*/%)	Any adverse outcome (*N*/%)	Adjusted OR	95% confidence limits
	*N *= 471	*N *= 47		
Maternal age < 25	6/1.3%	0/0.0%		
Maternal age ≥ 35	123/26.1%	12/25.5%	1.02	0.46–2.22
Maternal smoking	80/17.0%	6/12.8%	0.54	0.20–1.45
Nulliparous	232/49.3%	14/29.8%	0.43	0.18–0.99
Multipara ≥ 3 deliveries	61/13.0%	9/19.1%	1.31	0.39–4.44
BMI ≥ 30	67/14.2%	6/12.8%	0.16	0.02–1.28
BMI ≥ 35	18/3.8%	5/10.6%	32.19	2.97–348.65
History of induced abortion	72/15.3%	10/21.3%	1.60	0.69–3.71
History of miscarriage	111/23.6%	14/29.8%	1.03	0.48–2.18
History of cesarean section	72/15.3%	15/31.9%	1.79	0.79–4.06
Assisted reproduction technology	20/4.2%	0/0.0%		
Diabetes mellitus type I	24/5.1%	1/2.1%	2.53	0.27–24.17
Diabetes mellitus type II	3/0.6%	0/0.0%		
Gestational diabetes	43/9.1%	3/6.4%	1.66	0.40–6.89
Pre-eclampsia/High blood pressure	74/15.7%	2/4.3%	0.21	0.05–0.96
Oligohydramnios	12/2.5%	7/14.9%	6.50	2.00–21.11
Placenta previa	6/1.3%	0/0.0%	0.55	0.26–1.17
PPROM	152/32.3%	12/25.5%	0.55	0.26–1.17
Any congenital anomaly	19/4.0%	8/17.0%	4.50	1.56–12.96
Induced delivery	11/2.3%	3/6.4%	2.13	0.44–10.36
Epidural anesthesia in vaginal birth	25/26.3%	4/25.0%	1.48	0.47–4.63
Gestational age at delivery mean in days	216/10	210/10		
Fetal growth restriction (birth weight < 2SD)	6/1.3%	2/4.3%	5.89	1.00–34.74
Neonatal sex (female)	218/46.3%	17/36.2%	0.74	0.37–1.48

*BMI* body mass index, *PPROM* preterm premature rupture of membranes

In late preterm deliveries, the adverse outcomes became less frequent, as in 33 to 36 gestational weeks, only 47 out of 1544 deliveries (3.0%) had an adverse outcome. Significant risk factors for adverse outcomes in late preterm breech deliveries in our study were maternal smoking (aOR 2.29, CI 1.12–4.72), oligohydramnios (aOR 19.06, CI 7.15–50.85), epidural anesthesia in vaginal birth (aOR 2.44, CI 1.19–5.01), and fetal growth restriction (aOR 12.27, CI 2.81–53.66) (Table 3).

**Table 3:** Unadjusted and adjusted odds ratios for risk factors for adverse outcomes in 33 + 0 to 36 + 6 weeks of gestational age fetuses in breech presentations 2004–2018 in Finland

33–36 weeks of gestation	No adverse outcomes (*N*/%)	Any adverse outcome (*N*/%)	Adjusted OR	95% confidence limits
	*N *= 1497	*N *= 47		
Maternal age < 25	31/2.1%	1/2.1%	0.49	0.05–4.74
Maternal age ≥ 35	362/24.2%	11/23.4%	0.94	0.44–2.02
Maternal smoking	227/15.2%	14/29.8%	2.29	1.12–4.72
Nulliparous	872/58.2%	22/46.8%	0.87	0.42–1.83
Multipara ≥ 3 deliveries	116/7.7%	7/14.9%	1.42	0.43–4.68
BMI ≥ 30	155/10.4%	8/17.0%	0.92	0.25–3.35
BMI ≥ 35	57/3.8%	5/10.6%	2.88	0.54–15.19
History of induced abortion	174/11.6%	7/14.9%	0.98	0.39–2.44
History of miscarriage	355/23.7%	10/21.3%	0.69	0.31–1.51
History of cesarean section	172/11.5%	8/17.0%	1.82	0.71–4.63
Assisted reproduction technology	61/4.1%	0/0.0%		
Diabetes mellitus type I	24/1.6%	1/2.1%	0.43	0.05–3.56
Diabetes mellitus type II	3/0.2%	1/2.1%	0.11	0.01–1.39
Gestational diabetes	153/10.2%	5/10.6%	0.99	0.36–2.77
Pre-eclampsia/High blood pressure	106/7.1%	1/2.1%	0.20	0.03–1.60
Oligohydramnios	23/1.5%	8/17.0%	19.06	7.15–50.85
Placenta previa	20/1.3%	1/2.1%	2.13	0.26–17.41
PPROM	406/27.1%	9/19.1%	0.73	0.33–1.61
Any congenital anomaly	111/7.4%	4/8.5%	0.89	0.27–2.99
Induced delivery	123/8.2%	7/14.9%	1.38	0.51–3.71
Epidural anesthesia in vaginal birth	250/42.6%	15/55.6%	2.44	1.19–5.01
Gestational age at delivery mean in days	246/9	249/7		
Fetal growth restriction (birth weight < 2SD)	11/0.7%	3/6.4%	12.27	2.81–53.66
Neonatal sex (female)	703/47. 0%	26/55.3%	1.39	0.74–2.62

*BMI* body mass index, *PPROM* preterm premature rupture of membranes

## Discussion

Our study shows that for each subcategory of preterm birth, there are different risk factors for adverse neonatal outcomes in planned vaginal preterm breech delivery. In extremely preterm breech deliveries (24 + 0 to 27 + 6 weeks) PPROM was associated with adverse neonatal outcomes. In very preterm breech deliveries (28 + 0 to 32 + 6 weeks) severe maternal obesity, oligohydramnios, congenital anomalies, and fetal growth restriction were associated with a higher risk of adverse neonatal outcome. In moderate to late preterm breech deliveries (33 + 0 to 36 + 6 weeks) maternal smoking, oligohydramnios, epidural anesthesia, and fetal growth restriction were identified as risk factors.

Oligohydramnios was found to increase the adverse outcomes 6.5-fold for very preterm and 19-fold for moderate to late preterm breech deliveries in our study. The results are supported by the previous literature that has shown oligohydramnios to be a risk factor for adverse perinatal outcomes in term breech pregnancies [8]. Oligohydramnios is linked to diminished fetal movements, compression of the umbilical cord, insufficiency of the placental as well as fetal aspiration of meconium [8, 22, 23]. Besides, previous studies have shown that a low amniotic fluid amount is linked with lower Apgar scores, a higher risk of neonatal acidosis and cesarean section as the mode of delivery due to fetal distress [22].

Fetal growth restriction in term is a well-known contraindication for vaginal breech delivery, since it is indisputably linked to severe adverse perinatal outcomes such as hypoxic injuries and even neonatal death [8, 21]. Our results were coherent; fetal growth restriction (< − 2SD/IUGR) in planned vaginal breech delivery increased remarkably the risk for perinatal morbidity also in very preterm (sixfold) and in moderate to late preterm (12-fold) deliveries. Growth restricted fetuses might suffer more easily from distress during labor, and potential entrapment of the head during vaginal labor might increase the risks. Our study showed that smoking increased adverse outcomes in 33 to 36 weeks of gestation. Smoking is a major risk factor for intrauterine growth restriction and it is associated with obstetric complications such as placental abruption and placenta previa, as well as preterm birth itself [24, 25].

Maternal obesity was shown as a risk for neonatal health in very preterm breech deliveries. The risk for adverse outcome increased 32-fold, if women with a BMI above 35 attempted a trial of vaginal delivery among very preterm pregnancies. Antenatal and intrapartum obstetric complications, as well as perinatal morbidity and mortality, are known to be increased in obese mothers [7, 26, 27]. Maternal obesity is associated with preterm delivery itself, instrumental delivery, and cesarean section as a mode of birth [26, 27]. Also, evaluation of fetal wellbeing might be more difficult in obese women [26]. Our personal opinion is that the massive obesity has an effect on the birth channel and that a very preterm fetus is not able to path through the birth channel smoothly, through excessive soft tissue resistance.

One of the essential findings of our study was that the induction of labor did not increase the risks in preterm breech deliveries (24–27 weeks of gestation: aOR 0.50, *p* value 0.787, 28–32 weeks of gestation: aOR 2.13, *p* value 0.103, 33–36 weeks of gestation: aOR 1.38, *p* value 0.105). Contrary findings were found in a recent meta-analysis (2018) as induction of term breech labor was found to increase the risk of perinatal morbidity and cesarean sections [28]. However, Macharey et al. found no association between induction of term breech labor and neonatal morbidity, but the rate of vaginal deliveries was remarkably lower if term breech labor was induced compared to the spontaneous breech deliveries [29]. Induction of term labor in breech presentation was established as safe as planned cesarean delivery also in observational prospective study 2019 [30].

Our results showed a connection between epidural anesthesia in vaginal breech labor and adverse outcomes in moderate to late preterm deliveries. Earlier studies have already shown a connection between epidural anesthesia and prolonged labor in term breech deliveries [31]. Furthermore, epidural anesthesia during labor is associated with increased augmentation with oxytocin and over twofold higher risk of adverse outcomes in term breech deliveries [8, 31]. It has been speculated whether adverse outcomes are due to the fact that epidural anesthesia is more used in prolonged labors or epidural anesthesia itself increases the duration of labor [8, 31].

Interestingly, in our study pre-eclampsia or high blood pressure decreased the odds of adverse outcomes in very preterm breech deliveries. However, pre-eclampsia is a well-identified risk factor for maternal and neonatal mortality and morbidity [32]. Pre-eclampsia is associated with intrauterine growth restriction and congenital anomalies [32–34], in which both conditions increased the adverse perinatal outcomes in our study among very preterm deliveries. Our contradictory results might be explained by the fact that the small number of cases may not have had enough power to detect differences between the groups. Besides, maternal obstetric risk factors such as pre-eclampsia or high blood pressure may be considered as a contraindication for vaginal delivery and thus these women have more often a planned cesarean section as mode of delivery. These circumstances might cause bias in the results [35].

Other authors have shown primiparous women to have more adverse perinatal outcomes [36]. Furthermore, primiparity is linked to low birth weight in term pregnancies [37], and in our results, fetal growth restriction in preterm breech deliveries seemed to increase the risks. Nevertheless, primiparity in preterm breech deliveries was not found as a risk for perinatal morbidity in our study, and in contrary, primiparity appeared as a protective factor in very preterm vaginal breech delivery. This finding may partly be explained that the mode of birth is more likely primary cesarean section in nulliparous women when the fetus is in a breech position.

 Congenital anomalies in term breech pregnancies are known risks for perinatal morbidity and mortality [7, 9, 38], and our study showed similar results among very preterm breech fetuses. In addition, other studies have linked oligohydramnios, fetal growth restriction, maternal obesity, and high blood pressure to increased congenital anomalies [24, 27, 39], and as shown before, these factors were risks for adverse outcomes in our study as well. However, in many cases of severe congenital anomalies, vaginal delivery is favorable also in breech deliveries to minimize maternal morbidity [40].

In extremely preterm breech deliveries over 30% had adverse outcomes. In this group the only risk factor found for adverse outcomes was PPROM. The extremely preterm delivery itself is a major risk for short-term neonatal morbidity [41], and this fact may be the reason why we could not identify more risk factors. Sephton S showed PPROM to be associated with a significant risk of neonatal morbidity partly due to infections and placental abruptions [42–44]. Preterm fetuses with PPROM and born vaginally may not tolerate the contractions during labor or the compression when descending in the birth canal [45]. Preterm breech deliveries complicated with PPROM may also have more difficulties with the delivery of the aftercoming head [46]. In a Cochrane review (2017) Bond and colleagues pointed out that despite PPROM before 37 weeks’ gestation and without contraindications, expectant management in careful evaluation is associated with good neonatal outcomes [47].

Our study offers essential information about the risks of adverse outcomes in preterm breech deliveries. This is the first study that was able to identify risk factors for adverse neonatal outcomes in planned vaginal preterm breech delivery. Understanding the risks for adverse outcomes is essential for the decision-making on the mode of delivery when treating preterm breech deliveries. Obstetricians can now select for preterm breech presentation those women who should give birth by cesarean section in any case. Some of the risk factors like oligohydramnios, congenital anomalies, and fetal growth restriction were similar as in planned vaginal breech delivery at term [8, 21], but others, like severe maternal obesity and PPROM, were not known as risk factors for breech deliveries overall. This study is a unique population-based case–control study of the subject and offers valuable information for decision-making when treating preterm breech deliveries.

There are, however, few limitations in our study. Designed as a retrospective trial, we are exposed to the possibility of a typical bias of retrospective case–control studies. Additionally, data were restricted to the information of the data bank. In a few risk factors, we might have lacked statistical power to detect the risks of adverse outcomes, as there were only a few patients in the group. Nevertheless, our study was population-based and included over 2300 vaginal preterm breech deliveries from 14 years. Because there are no private birth hospitals in Finland, the treatment of the deliveries are homogenous and comparative.

## Conclusion

 We recommend a planned cesarean section for women with severe maternal obesity (BMI > 35), oligohydramnios, or fetal growth restriction in very preterm breech deliveries (28 + 0 to 32 + 6 weeks) and for women with oligohydramnios or a fetus with fetal growth restriction (< 2 SD) in moderate to late preterm breech deliveries (33 + 0 to 36 + 6 weeks).

## Data Availability

The Finnish register data have been given for this specific study, and the data cannot be shared without authorization from the register keepers. More information on the authorization application to researchers who meet the criteria for access to confidential data can be found at Findata, the Health and Social Data Permit Authority: https://www.findata.fi/en/.

## Abbreviations

ICD-10: International Statistical Classification of Diseases and Related Health Problems 10th Revision
BMI: Body mass index
PPROM: Preterm premature rupture of membranes
SD: The second standard deviation
WHO: World Health Organization
OR: Crude odds ratio
Cl: Confidence interval
aOR: Adjusted odds ratio
